# Supplementary material for: Meta-Analysis Reveals Challenges and Gaps for Genome-to-Phenome Research Underpinning Plant Drought Response
Source: Int J Mol Sci. 2022 Oct 14;23(20):12297. doi: 10.3390/ijms232012297 (PMC9602940; doi:10.3390/ijms232012297)

Supplemental Text S1

To understand the adaptive capacity of plant populations towards drought, we must recognize the physiological risks for plants experiencing water deficit. Water is an essential resource for plants to maintain structure, perform photosynthesis, grow, and survive to reproduce. Depending on the length and severity of drought conditions, plants are susceptible to mortality through complex interactions between hydraulic failure, carbon starvation, and biotic stressors ([71]; Fig. S4). When plants experience drought, dissolved air within sap or plant water can expand (i.e., cavitation), causing air bubbles to fill xylem vessels and tracheids [69]. Such 'breaks' in the water column, also known as embolisms, prevents the essential transport of water between roots and leaves, leading to hydraulic failure. While some plants can shift their physiology to maximize water retention during drought and refill vessels and tracheids upon rewatering [72], severe droughts or droughts of long duration can lead to desiccation and mortality by xylem cavitation, embolism, and hydraulic failure [69,71]. Hydraulic failure is a leading factor in plant drought mortality in plants, with examples in crop species [73] and natural plant communities [74,75]. Additionally, other stressors can act antagonistically with hydraulic failure to increase plant mortality under drought conditions.

Physiological responses to prevent hydraulic failure can lead to another risk to plant survival during drought: carbon starvation. When plants detect water deficits, stomata close in order to reduce transpiration and avoid a marked decline in plant water potential. However, this response subsequently eliminates exchange gas, photosynthesis, and the production of carbohydrates to maintain plant metabolism, growth, and defence against disease [76]. Carbon starvation—when carbohydrate use is higher than supply and production—is described as a slow process leading to mortality, but can also work in conjunction with hydraulic failure to contribute

to increased plant mortality due to drought [71,76]. For example, a study of *Pinus edulis* Engelm. by Sevanto et al. [77] illustrates how trees that undergo mild, but sustained drought experience compounded effects from both hydraulic failure and carbon starvation. In this study, lack of hydraulic conductance is hypothesized to exacerbate carbon starvation by impairing the ability of phloem cells to transport carbohydrates. In addition, lack of carbohydrate reserves diminishes the ability of trees to refill its tracheids after cavitation [77].

In addition to interactions between carbon starvation and hydraulic failure, these abiotic and physiological drought stressors can make plants susceptible to a third risk: biotic stresses, such as pathogen attach and herbivory [78]. Some plants can initiate pathogen defence systems during mild drought stress, making it possible to protect against infection and disease [79]. However, during severe drought stress, plants usually have weakened defence systems making them more susceptible to viral, bacterial, and fungal attack [80]. In addition to pathogens, plants are also more likely to experience herbivory during drought. This can be explained by these organisms seeking water sources during drought conditions, or by plants having reduced defences against herbivores during severe drought [78]. Some herbivorous insects may also be the vectors of plant pathogens, creating complex tripartite biotic interactions between hosts, vectors, and pathogens [81]. These biotic and abiotic stressors associated with drought are interconnected and plants have developed sophisticated and coordinated adaptations to avoid drought-related mortality.

To reduce the risk of drought mortality, plants have developed diverse and complex physiological responses across life history stages, such as seedling establishment and recruitment and the survival and reproduction of mature plants, to survive drought [82,83]. Drought response strategies among plants are diverse, but are broadly grouped into three categories: detection,

resistance, and recovery (Fig. S3). Researchers are still characterizing the initial drought detection mechanisms, but it likely begins with osmosensors that sense hydraulic or osmotic shifts on a cellular level [84,85]. This detection process leads to signalling cascades via protein kinases and changes in Ca^{2+} concentrations, resulting in the synthesis of phytohormones that help regulate drought responses ([64,86,87]. While the hormone abscisic acid (ABA) has been largely associated with many facets of drought response (e.g., stomatal activity and increase in the root to shoot ratio), other hormones including auxins, ethylene (ET), jasmonic acid (JA), and salicylic acid (SA) are also associated with biotic and abiotic stress response pathways as well [64,81,88,89].

Drought resistance in plants is determined by their ability to survive and grow under soil water deficits, and can be further classified into three strategies: drought escape (i.e., initiating reproduction earlier, to ensure fitness), drought avoidance or postponement (i.e., maximising water capacity through closing stomata, investing in root growth, osmotic adjustment, increasing tissue storage, and/or building wax cuticles), and drought tolerance (i.e., regulating osmotic interactions, photosynthesis, anti-oxidant production, and tissue repair to withstand water deficit; [9,42,65,90]. These resistance strategies are not necessarily mutually exclusive and can work synergistically to reduce fitness [64]. For example, drought avoidance tactics like stomatal closing can reduce photosynthesis, and trigger the production and accumulation of reactive oxygen species (ROS) in cell organelles including chloroplasts, peroxisomes, and mitochondria [91]. While minimal amounts of ROS are normal for plants, and can be helpful for drought signalling, large amounts of ROS can lead to cellular death. In response to this secondary stressor, tolerance strategies including the production of antioxidants can ‘scavenge’ ROS so that they function to signal drought response, as opposed to causing cellular death [65]. Drought

avoidance and tolerance strategies, if successful, can prevent hydraulic failure and subsequently reduce mortality [11,92,93].

Drought recovery, or the ability of plants to refill xylem vessels, repair tissues, re-open stomata, and resume photosynthesis following re-watering, is a crucial component of the drought response process [94]. Drought recovery for some plant species can be rapid, depending on the length and severity of the drought. However, some studies have revealed that some plants experience a stress 'memory' following sustained or repeated droughts, causing stomata to remain semi-closed even after re-watering [95,96]. Similarly, xylem vessels and tracheids can experience refilling fatigue after repeated cavitation-refilling cycles leading to reduced xylem conductivity due to cell damage (e.g., pit membrane ruptures, or stretching of microfibril mesh; [72]). The ability of plants to withstand drought can vary between taxa [97] and among individuals within populations [98]. Linking this phenotypic variation to genetic mechanisms that underpin drought tolerance (i.e., genome-to-phenome or G2P interactions) will be critical to understanding the adaptive capacity of plants towards a changing climate.

The complex adaptive response of plants towards drought is likely controlled by a suite of environmental, biotic, molecular, and genomic mechanisms; these include genomic-based adaptive response to environmental stressors (i.e., adaptive plasticity; [42]), and genetic variation at loci contributing to drought adaptations (i.e., functional diversity; reviewed in [90]), environmentally-induced change in gene expression (i.e., epigenetics; [99]), and changes in microbiome composition and interactions [100].

Supplemental Literature Cited

71. McDowell, N.; Pockman, W.T.; Allen, C.D.; Breshears, D.D.; Cobb, N.; Kolb, T.; Plaut, J.; Sperry, J.; West, A.; Williams, D.G.; et al. Mechanisms of Plant Survival and Mortality during Drought: Why Do Some Plants Survive While Others Succumb to Drought? *New Phytol.* **2008**, *178*, 719–739, doi:10.1111/j.1469-8137.2008.02436.x.
72. Hacke, U.G.; Stiller, V.; Sperry, J.S.; Pittermann, J.; McCulloh, K.A. Cavitation Fatigue. Embolism and Refilling Cycles Can Weaken the Cavitation Resistance of Xylem. *Plant Physiol.* **2001**, *125*, 779–786, doi:10.1104/pp.125.2.779.
73. Li, Y.; Sperry, J.S.; Shao, M. Hydraulic Conductance and Vulnerability to Cavitation in Corn (*Zea mays* L.) Hybrids of Differing Drought Resistance. *Environ. Exp. Bot.* **2009**, *66*, 341–346, doi:10.1016/j.envexpbot.2009.02.001.
74. Vilagrosa, A.; Bellot, J.; Vallejo, V.R.; Gil-Pelegrín, E. Cavitation, Stomatal Conductance, and Leaf Dieback in Seedlings of Two Co-Occurring Mediterranean Shrubs during an Intense Drought. *J. Exp. Bot.* **2003**, *54*, 2015–2024, doi:10.1093/jxb/erg221.
75. Choat, B.; Jansen, S.; Brodribb, T.J.; Cochard, H.; Delzon, S.; Bhaskar, R.; Bucci, S.J.; Feild, T.S.; Gleason, S.M.; Hacke, U.G.; et al. Global Convergence in the Vulnerability of Forests to Drought. *Nature* **2012**, *491*, 752–755, doi:10.1038/nature11688.
76. McDowell, N.G. Mechanisms Linking Drought, Hydraulics, Carbon Metabolism, and Vegetation Mortality. *Plant Physiol.* **2011**, *155*, 1051–1059, doi:10.1104/pp.110.170704.
77. Sevanto, S.; Mcdowell, N.G.; Dickman, L.T.; Pangle, R.; Pockman, W.T. How Do Trees Die? A Test of the Hydraulic Failure and Carbon Starvation Hypotheses. *Plant, Cell Environ.* **2014**, *37*, 153–161, doi:10.1111/pce.12141.
78. Jactel, H.; Petit, J.; Desprez-Loustau, M.L.; Delzon, S.; Piou, D.; Battisti, A.; Koricheva, J. Drought Effects on Damage by Forest Insects and Pathogens: A Meta-Analysis. *Glob. Chang. Biol.* **2012**, *18*, 267–276, doi:10.1111/j.1365-2486.2011.02512.x.
79. Qiu, Y.; Yu, D. Over-Expression of the Stress-Induced OsWRKY45 Enhances Disease Resistance and Drought Tolerance in Arabidopsis. *Environ. Exp. Bot.* **2009**, *65*, 35–47, doi:10.1016/j.envexpbot.2008.07.002.
80. Ramegowda, V.; Senthil-Kumar, M. The Interactive Effects of Simultaneous Biotic and Abiotic Stresses on Plants: Mechanistic Understanding from Drought and Pathogen Combination. *J. Plant Physiol.* **2015**, *176*, 47–54, doi:10.1016/j.jplph.2014.11.008.
81. Szczepaniec, A.; Finke, D. Plant-Vector-Pathogen Interactions in the Context of Drought Stress. *Front. Ecol. Evol.* **2019**, *7*, 1–7, doi:10.3389/fevo.2019.00262.
82. Kerr, K.L.; Meinzer, F.C.; Mcculloh, K.A.; Woodruff, D.R.; Marias, D.E. Expression of Functional Traits during Seedling Establishment in Two Populations of Pinus Ponderosa from Contrasting Climates. *Tree Physiol.* **2015**, *35*, 535–548, doi:10.1093/treephys/tpv034.
83. Adams, H.D.; Zeppel, M.J.B.; Anderegg, W.R.L.; Hartmann, H.; Landhäusser, S.M.; Tissue, D.T.; Huxman, T.E.; Hudson, P.J.; Franz, T.E.; Allen, C.D.; et al. A Multi-Species Synthesis of Physiological Mechanisms in Drought-Induced Tree Mortality. *Nat. Ecol. Evol.* **2017**, *1*, 1285–1291, doi:10.1038/s41559-017-0248-x.
84. Streck, N.A. Do We Know How Plants Sense a Drying Soil? *Ciência Rural* **2004**, *34*, 581–584, doi:10.1590/s0103-84782004000200039.
85. Robbins, N.E.; Dinneny, J.R. The Divining Root: Moisture-Driven Responses of Roots at the Micro- and Macro-Scale. *J. Exp. Bot.* **2015**, *66*, 2145–2154, doi:10.1093/jxb/eru496.
86. Xiong, L.; Schumaker, K.S.; Zhu, J.-K. Cell Signaling during Cold, Drought, and Salt Stress. *Plant Cell* **2002**, *14*, S165–S183, doi:10.1105/tpc.000596.
87. Zhu, J.K. Salt and Drought Stress Signal Transduction in Plants. *Annu. Rev. Plant Biol.* **2002**, *53*, 247–273, doi:10.1146/annurev.arplant.53.091401.143329.
88. Jakab, G.; Ton, J.; Flors, V.; Zimmerli, L.; Métraux, J.P.; Mauch-Mani, B. Enhancing Arabidopsis Salt and Drought Stress Tolerance by Chemical Priming for Its Abscisic Acid Responses. *Plant Physiol.* **2005**, *139*, 267–274, doi:10.1104/pp.105.065698.
89. Zhang, J.; Jia, W.; Yang, J.; Ismail, A.M. Role of ABA in Integrating Plant Responses to Drought and Salt Stresses. *F. Crop. Res.* **2006**, *97*, 111–119, doi:10.1016/j.fcr.2005.08.018.
90. Moran, E.; Lauder, J.; Musser, C.; Stathos, A.; Shu, M. The Genetics of Drought Tolerance in Conifers. *New Phytol.* **2017**, *216*, 1034–1048, doi:10.1111/nph.14774.
91. Cruz De Carvalho, M.H. Drought Stress and Reactive Oxygen Species: Production, Scavenging and Signaling. *Plant Signal. Behav.* **2008**, *3*, 156–165, doi:10.4161/psb.3.3.5536.
92. Munné-Bosch, S.; Alegre, L. Die and Let Live: Leaf Senescence Contributes to Plant Survival under Drought Stress. *Funct. Plant Biol.* **2004**, *31*, 203–216, doi:10.1071/FP03236.
93. Blackman, C.J.; Brodribb, T.J.; Jordan, G.J. Leaf Hydraulics and Drought Stress: Response, Recovery and Survivorship in Four Woody Temperate Plant Species. *Plant, Cell Environ.* **2009**, *32*, 1584–1595, doi:10.1111/j.1365-3040.2009.02023.x.
94. Chen, D.; Wang, S.; Cao, B.; Cao, D.; Leng, G.; Li, H.; Yin, L.; Shan, L.; Deng, X. Genotypic Variation in Growth and Physiological Response to Drought Stress and Re-Watering Reveals the Critical Role of Recovery in Drought Adaptation in Maize Seedlings. *Front. Plant Sci.* **2016**, *6*, 1–15, doi:10.3389/fpls.2015.01241.
95. Goh, C.H.; Gil Nam, H.; Shin Park, Y. Stress Memory in Plants: A Negative Regulation of Stomatal Response and Transient Induction of Rd22 Gene to Light in Abscisic Acid-Entrained Arabidopsis Plants. *Plant J.* **2003**, *36*, 240–255, doi:10.1046/j.1365-313X.2003.01872.x.
96. Virlouvet, L.; Fromm, M. Physiological and Transcriptional Memory in Guard Cells during Repetitive Dehydration Stress. *New Phytol.* **2015**, *205*, 596–607, doi:10.1111/nph.13080.

97. Jacobsen, A.L.; Pratt, R.B.; Davis, S.D.; Ewers, F.W. Cavitation Resistance and Seasonal Hydraulics Differ among Three Arid Californian Plant Communities. *Plant, Cell Environ.* **2007**, *30*, 1599–1609, doi:10.1111/j.1365-3040.2007.01729.x.
98. Luo, N.; Liu, J.; Yu, X.; Jiang, Y. Natural Variation of Drought Response in Brachypodium Distachyon. *Physiol. Plant.* **2011**, *141*, 19–29, doi:10.1111/j.1399-3054.2010.01413.x.
99. Gutzat, R.; Mittelsten Scheid, O. Epigenetic Responses to Stress: Triple Defense? *Curr. Opin. Plant Biol.* **2012**, *15*, 568–573, doi:10.1016/j.pbi.2012.08.007.
100. de Vries, F.T.; Griffiths, R.I.; Knight, C.G.; Nicolitch, O.; Williams, A. Harnessing Rhizosphere Microbiomes for Drought-Resilient Crop Production. *Science* **2020**, *368*, 270–274, doi:10.1126/science.aaz5192.

Supplemental Figures

Figure S1. Occurrence points for each of the species represented in all abstracts that occur in hyper-arid. This set comprises 32 species out of 106 non-model plants that were queried for this analysis.

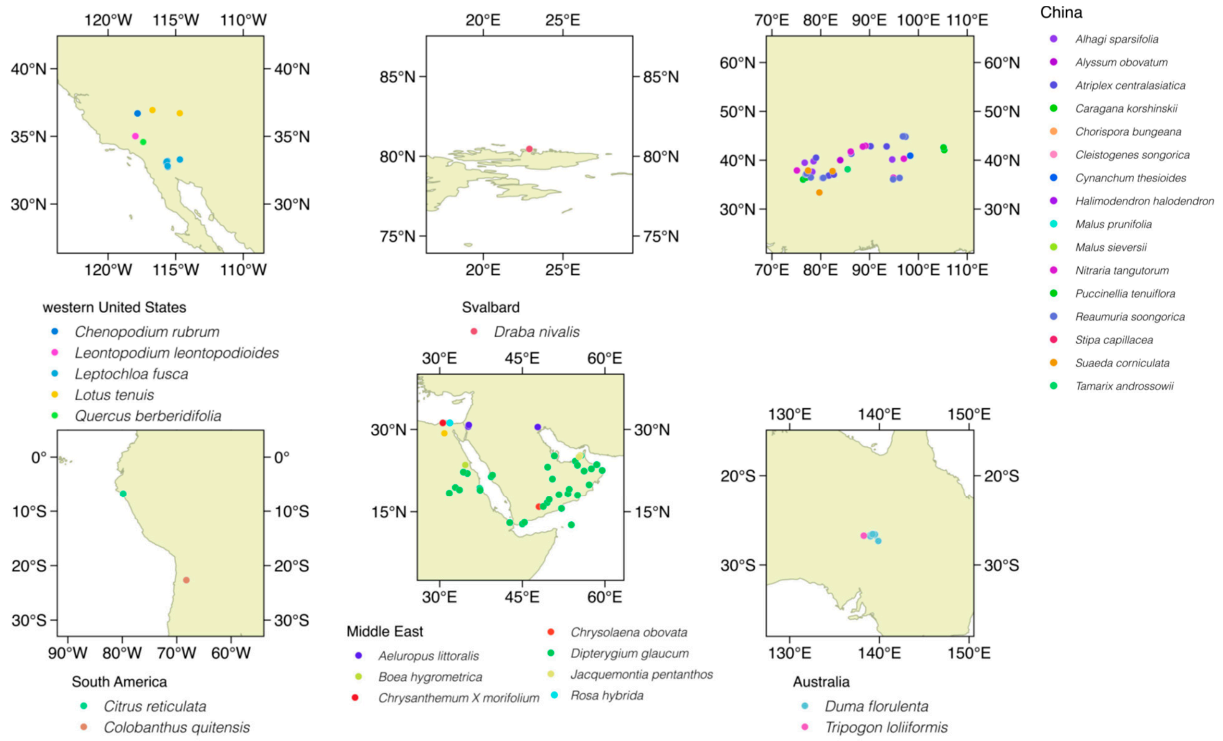

Figure S2. Total number of abstracts per year associated with gene or taxon data acquired accessed via text mining, database mining.

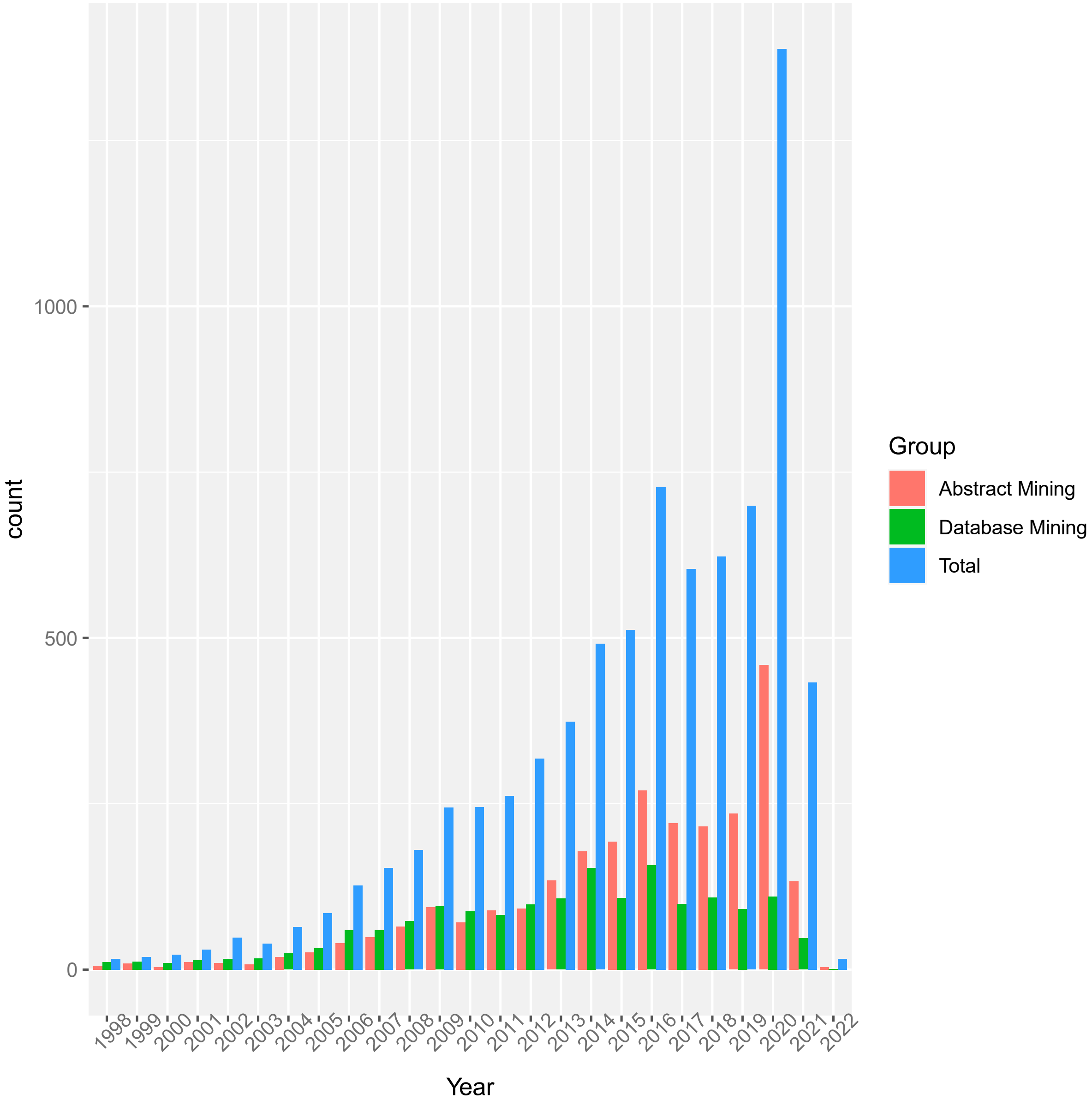

Figure S3. Diagram modified from McDowell et al. (2008) [71], postulating the risk of plant death during drought as a function of drought intensity (blue), duration (red), and susceptibility to herbivory and disease (grey dots).

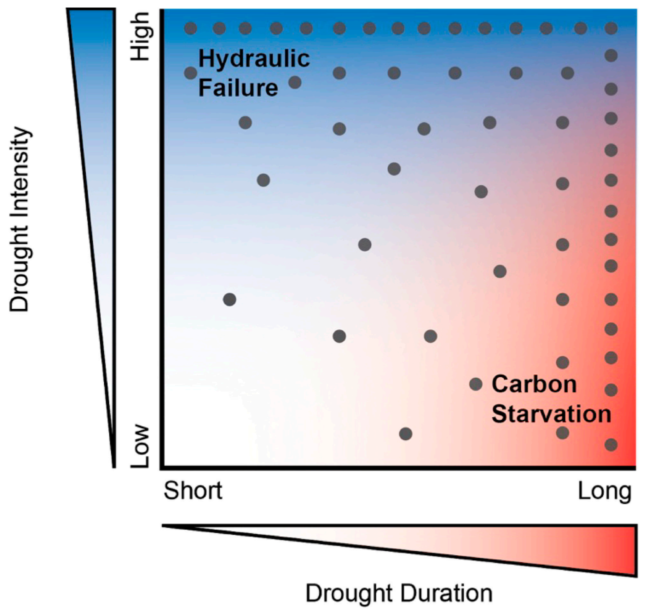

Figure S4. Categories for drought response strategies and examples of responses within each category.

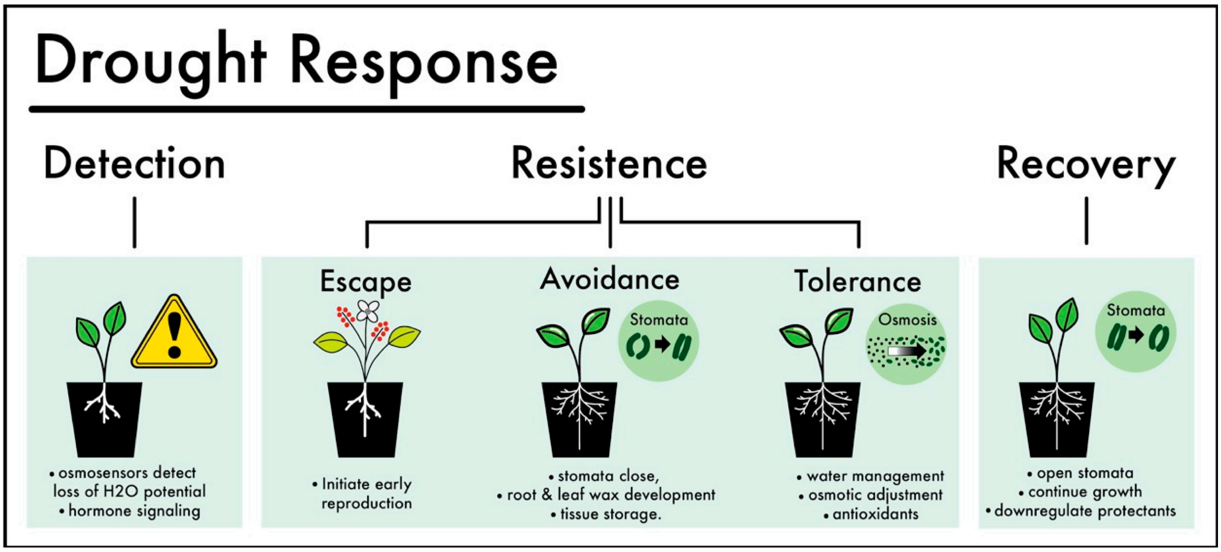

Supplement: Supplementary file 1 [file ijms-23-12297-s001.zip › Supplemental Text S1.pdf]
